# Effect of Time on Human Muscle Outcomes During Simulated Microgravity Exposure Without Countermeasures—Systematic Review

**DOI:** 10.3389/fphys.2019.01046

**Published:** 2019-08-16

**Authors:** Andrew Winnard, Jonathan Scott, Nathan Waters, Martin Vance, Nick Caplan

**Affiliations:** ^1^Faculty of Health and Life Sciences, Northumbria University, Newcastle upon Tyne, United Kingdom; ^2^Space Medicine Office, European Astronaut Centre, Cologne, Germany

**Keywords:** muscle, microgravity, spaceflight, deconditioning, astronaut

## Abstract

**Background:** Space Agencies are planning human missions beyond Low Earth Orbit. Consideration of how physiological system adaptation with microgravity (μG) will be managed during these mission scenarios is required. Exercise countermeasures (CM) could be used more sparingly to decrease limited resource costs, including periods of no exercise. This study provides a complete overview of the current evidence, making recommendations on the length of time humans exposed to simulated μG might safely perform no exercise considering muscles only.

**Methods:** Electronic databases were searched for astronaut or space simulation bed rest studies, as the most valid terrestrial simulation, from start of records to July 2017. Studies were assessed with the Quality in Prognostic Studies and bed rest analog studies assessed for transferability to astronauts using the Aerospace Medicine Systematic Review Group Tool for Assessing Bed Rest Methods. Effect sizes, based on no CM groups, were used to assess muscle outcomes over time. Outcomes included were contractile work capacity, muscle cross sectional area, muscle activity, muscle thickness, muscle volume, maximal voluntary contraction force during one repetition maximum, peak power, performance based outcomes, power, and torque/strength.

**Results:** Seventy-five bed rest μG simulation studies were included, many with high risk of confounding factors and participation bias. Most muscle outcomes deteriorated over time with no countermeasures. Moderate effects were apparent by 7–15 days and large by 28–56 days. Moderate effects (>0.6) became apparent in the following order, power and MVC during one repetition maximum (7 days), followed by volume, cross sectional area, torques and strengths, contractile work capacity, thickness and endurance (14 days), then muscle activity (15 days). Large effects (>1.2) became apparent in the following order, volume, cross sectional area (28 days) torques and strengths, thickness (35 days) and peak power (56 days).

**Conclusions:** Moderate effects on a range of muscle parameters may occur within 7–14 days of unloading, with large effects within 35 days. Combined with muscle performance requirements for mission tasks, these data, may support the design of CM programmes to maximize efficiency without compromising crew safety and mission success when incorporated with data from additional physiological systems that also need consideration.

## Introduction

### Rationale

Space Agencies are planning to transition from International Space Station (ISS) missions to Lunar missions including a crewed base from which to test and develop hardware and procedures required for the longer term goal of human Mars missions (Foing, 2016). It is well documented that exposure to microgravity (μG) during spaceflight causes adaptation in response to gravitational unloading, especially in the musculoskeletal, cardiovascular and neuro-vestibular systems (Buckey, 2006; Baker et al., 2008). The risks to mission success due to potential injury or reduced function due to periods of unmanaged adaptation before arriving at a remote location such as Moon or Mars, where medical teams may not be present on landing, need to be addressed (Gernand, 2004). Bed rest is often used as a controlled Earth-based environment for simulating the effects of spaceflight on humans to enable more cost-effective, higher quality and safer research into effects and medical management of adaptation (Pavy-Le Traon et al., 2007). While bed rest fails to remove a Gx (chest-to-back) loading vector, such studies are considered the most valid simulation method for many physiological systems (Adams et al., 2003; Pavy-Le Traon et al., 2007), except for weight bearing, tissue fluid redistributions and skin surface areas of compression (Hargens and Vico, 2016), and when conducted rigorously are likely to generate results transferable to astronauts (Higgins and Green, 2011;Winnard and Nasser, 2017b).

Based on bed rest research and previous spaceflight experience, the ISS provides astronauts with 2.5 h per day for exercise (including setup, stowage and hygiene) using a treadmill, cycle ergometer and resistance exercise device designed and adapted for μG (Trappe et al., 2009; Loehr et al., 2011). Several years of refining ISS exercise countermeasures (CM) has led to astronauts completing 6-month missions with, on average, little to no change in bone mass or cardiovascular capacity, although the efficacy seems to vary widely between individuals (Moore et al., 2014; English et al., 2015; Sibonga et al., 2015), while muscle adaptation appears to have become progressively smaller as exercise devices and prescriptions have improved (Smith et al., 2012; Moore et al., 2014; Ploutz-Snyder et al., 2015). However, the exercise devices currently aboard ISS will almost certainly be too large and too numerous for, and the exercise prescriptions place too great a demand on the consumables and environmental management systems available on, the vehicles and habitats currently planned for future exploration missions. For this reason, space agencies have started designing smaller, low energy and low vibration exercise devices (Brusco, 2016). However, decisions will need to be made regarding choice and/or development of an effective exercise CM programme to manage physiological adaptation that will occur during exploration missions. Considerations may include reducing the frequency of exercise as currently performed on ISS and potentially having longer periods of not performing any exercise. The duration of any such no exercise periods needs to be evidence based to balance any increase physiological risks to crew against gains in spacecraft and consumables impact.

### Objectives

The objectives of this review were to provide a complete summary of and synthesize the current space-related physiological evidence base and to inform decision making processes around muscle performance requirements, regarding operational CM, for exploration human space missions. Where data is lacking for any outcomes this will be highlighted as a gap or limited area of the current evidence base and used to provide a gap analysis commentary useful for future research priority setting. The aim is to aid space agencies in designing CM programmes, provide a complete summary of what muscle groups and outcomes have been assessed in the current evidence and highlight areas of minimal data or research gaps to guide future relevant research in this area. The NASA Risk Table for the Human Research Project highlights potential risks relating to spaceflight and shows the large scope of potential physiological systems that require reviewing to cover all elements of crew health and performance (National Aeronautics and Space Administration, 2019). As the scope is too large for a single review, it is suggested that the various systems be reviewed individually. Once a series of reviews has been complete a position statement summarizing across each system can provide a holistic overview. Therefore, this specific review investigated the rate at which muscle parameters change during simulated μG exposure, when no countermeasures are taken, to inform operational decisions regarding the possibility of using exercise CM programmes more sparingly for exploration missions, including the implementation of exercise “holidays” (i.e., a period of time within the mission when no exercise CM are employed). Conclusions of this review alone must be treated in a muscle context and need considering alongside other relevant health and performance components.

### Research Question

At what time point do people exposed to simulated μG while not performing CM reach a moderate or large effect on muscle health outcomes?

## Methods

### Study Design

The Cochrane Collaboration Guidebook (Higgins and Green, 2011) and preferred reporting items for systematic reviews and meta-analyses (PRISMA) were adhered to Moher et al. (2009). No external funding or research grants were received for this work.

#### Participants

The following inclusion criteria were employed. The target population was astronauts, however, as astronauts have taken part in space agency recommended exercise programmes to date, there was no inactive data available from this population. Therefore, healthy terrestrial adults, with no gender restrictions, taking part in μG analog bed rest studies, were included. Bed rest studies were the only terrestrial model included as they are considered the most valid ground based model for simulating human spaceflight for periods beyond a few minutes (Adams et al., 2003; Pavy-Le Traon et al., 2007). Therefore, to maintain the greatest level of transferability to astronauts and in keeping with our other systematic reviews only bed rest studies that stated they were simulating human spaceflight were considered. No clinical bed rest situations such as critical care were included as they would likely have confounding co-morbities and not transfer well to astronauts. All participants in the included bed rest studies were healthy at baseline, however, no exclusion was made relating to baseline level of physical condition beyond being healthy. Only control group data were relevant, therefore no inclusion criteria were based on interventions. Control groups had to be inactive and not undergo any type of intervention. Included studies had to report outcomes relating to muscles. For completeness of reporting the current state of the evidence base and avoid introducing selection bias, no exclusion was made based on type of outcome or amount of data. The evidence based led outcomes were determined from pre-scoping and the main review searches and were grouped for analysis as cross-sectional area, volume, shape, size, activity, power, performance and joint torque and forces at either a regional or global level. Included studies had to be randomized controlled trials (RCT), controlled clinical trials (CT), longitudinal, interrupted time series or before and after studies.

### Systematic Review Protocol

#### Search Strategy, Data Sources, Studies Sections, and Data Extraction

A range of relevant terms grouped by main search terms were constructed using Boolean logic (astronaut^*^, spaceflight, space flight, space^*^, weightless^*^, microgravity, micro gravity, bed-rest, bed rest, bed rest, dry immersion, muscle^*^, strength^*^) to search the following databases up to July 2017: Pubmed, CINAHL, Web of Science, NASA Technical Reports Server and The Cochrane Collaboration Library. No restrictions on type of bed rest or publication dates were applied, and due to the inability to use “Boolean logic” on the NASA Technical Reports Server, the strategy was adapted to keyword searches. The full search strategy is available in Table 1.

**Table 1:** Search strategy for database literature search.

**Search number**	**Term**	**Keywords in Boolean logic format**
1	Microgravity	“astronaut” OR “spaceflight OR “space flight OR “space*” OR “weightless*” OR “microgravity” OR “micro gravity”
2	Bed rest	“bed-rest” OR “bedrest” OR “bed rest” OR “dry immersion”
3	Muscle	“musc*” OR “strength*”
4	Combined	1 AND 2 AND 3

Initial screening was performed using abstracts and titles by two authors (MV and AW), blinded to each other's decisions, using Rayyan (https://rayyan.qcri.org/) (Ouzzani et al., 2016). Rayyan also automatically detects duplicate studies and data and all flagged potential duplication was assessed by agreement of three blinded authors. Where there was any disagreement whether the study met the inclusion criteria from initial screening the full text was obtained. A third author (NC) was used to resolve disagreements of included/excluded studies. An adapted version of the Aerospace Medicine Systematic Review Group (AMSRG) “Data extraction form,” version 2, July 2017 (AMSRG, 2018) was used by two authors (MV and NW) to extract data from each paper, and disagreements were discussed by three authors (AW, NW and MV) to reach consensus.

### Data Analysis

#### Quality Assessment

The Quality in Prognostic Studies (QUIPS) tool was used to assess risk of bias of all the included studies, with “H,” “M,” and “L” showing high, moderate and low risk, respectively, using pre-defined published definitions for each level (Hayden et al., 2013). Risk of bias results were used to comment on the current quality and completeness of the evidence base and do not change how studies were treated during analysis. As per published recommendations, only studies that were rated low risk of bias in all QUIPS domains were deemed as low risk overall (Hayden et al., 2013). The AMSRG “Tool for Assessing Bed Rest Methods” (Winnard and Nasser, 2017a,b) was used to assess the bed rest methodological quality, and transferability to astronaut populations, of all included studies, with “y” indicating the point was met, “n” not met, and “?” unclear. This is a relatively new tool, yet to be validated, that has been used in several other reviews (Richter et al., 2017; Winnard et al., 2017) and the development of the tool is explained in Winnard et al. (2017).

#### Main Analysis

Effect sizes (Hedges' g) were calculated between pre and post-bed rest values for each outcome individually without an overall pooled effect. Hedges' g was used to bias correct for the typically small sample sizes, as only control group data from μG simulation studies were eventually included. The reported data set that was as close to immediate pre and the end of bed rest was used for the analysis. No exclusion or analysis variation was made based on the individual study analysis methods. The pooled standard deviation for Hedges' g was calculated using the root mean square of the pre and post-group standard deviations. This version does not specifically include the sample size (n), preventing any complications that could arise from inflating n when both group means are from the same sample. Results were first sub-grouped by outcome measure type and then by muscle group before being listed in order of ascending days spent in simulated μG. Individual effects sizes were calculated and plotted in figures for each outcome at every time point where data were available. To enable a brief overview of the large data set to also be provided, an unweighted mean effect at each common time point within each muscle group was used to provide a summary result. This was only done when more than one study assessed the same outcome at the same time point. These statistics were chosen due to data being from the same sample rather than a separate intervention and control group, thus making a traditional weighted effects meta-analysis pooling inappropriate. Traditional meta-analysis assumes two different sets of individuals in each group (Higgins and Green, 2011) meaning a violation of underlying assumptions would have occurred if applied to this review. The summary unweighted mean, while being a less robust statistic, enabled an overarching overview to be reported in addition to each individual effect size, and overlaid on the figures, without violating statistical assumptions. Ninety-five percent confidence intervals were calculated for individual and unweighted group means. Readers should note that due to varying effect sizes across the various muscles and groupings, the effect size axis scale varies accordingly throughout the figures.

The point at which effects consistently reached a magnitude of 0.6 (moderate) or 1.2 (large) was highlighted as a time point when a worthwhile mechanistic change had occurred (Hopkins et al., 2009). Plots of all individual effects and 95% confidence interval tails, in order of ascending days in simulated μG, were overlaid with the mean effect and polynomial trend line of the mean effects. A polynomial trend allowed for the trend line to curve in case of progressively worsening, or plateauing patterns. In cases where data were lacking and varied (spanning more than one effect size cut off between data points), the trend line was highlighted as likely unreliable in the results section, meaning more data should be collected before a reliable trend can be established. The limited data sets are however still included for completeness of reporting the current state of the evidence base and highlight both minimal data areas and research gaps. The mean effect summary and trend line were only used to visually highlight the time point at which the mean effects passed the 0.6 and 1.2 magnitude point. A funnel plot of all the mean effects plotted against study size was used to show potential publication bias.

#### Sub Group Analysis

Ten sub groups were created based on the measurement methods units used for each for analysis as follows (with original units measured in): (1) contractile work capacity (J), (2) cross sectional area (mm^2^, cm^2^), (3) muscle activity (μV, mV, normalized), (4) muscle thickness (mm, cm), (5) muscle volume (cm^3^), (6) maximal voluntary contraction force during one repetition maximum (kg, N, Nm) (7), peak power (W), (8) performance based outcomes (including endurance time, jump power, force, velocity height and acceleration, sit to stand time, center of mass variation, and sprint time) (s, mm, cm, m, W/kg), (9) power (rad·s^−1^, m·s^−1^), and (10) torques and strength (Nm, ft-lb). Within each subgroup data were further sub-grouped for analysis by major muscle groups. For completeness of reporting, any measures that did not fit within major muscle groupings were grouped for analysis and reported as either “other lower limb,” “other trunk,” or “other upper limb” outcomes, to enable every outcome measure extracted from included studies to be reported in the results. The outcomes included in the “other” groupings are listed in the text.

## Results

### Study Selection, Characteristics, and Risk of Bias

**Figure 1:**
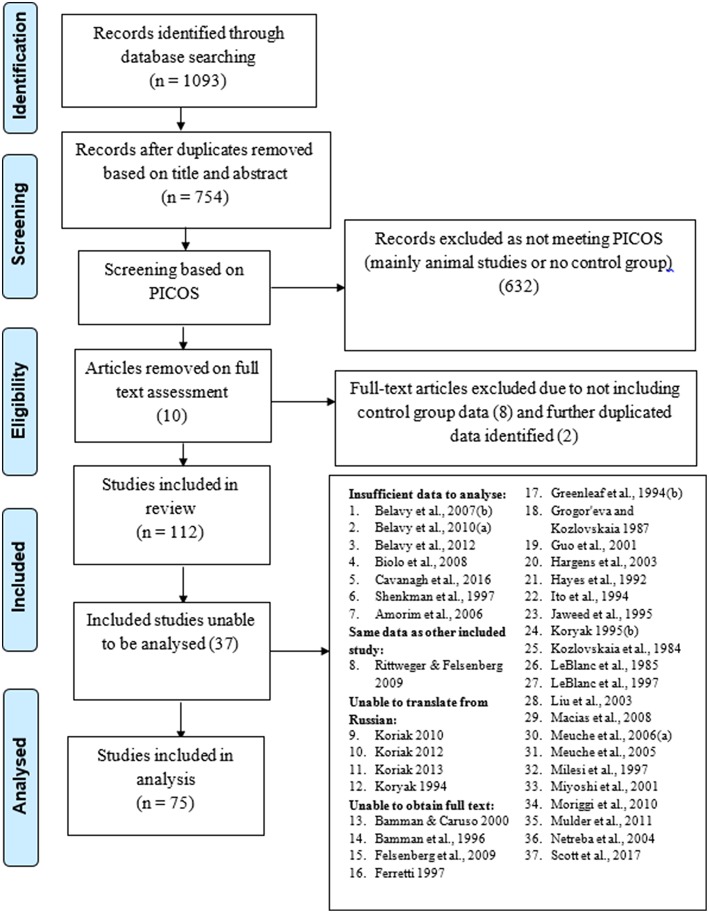
PRISMA flow diagram of inclusion/exclusion process.

**Figure 2:**
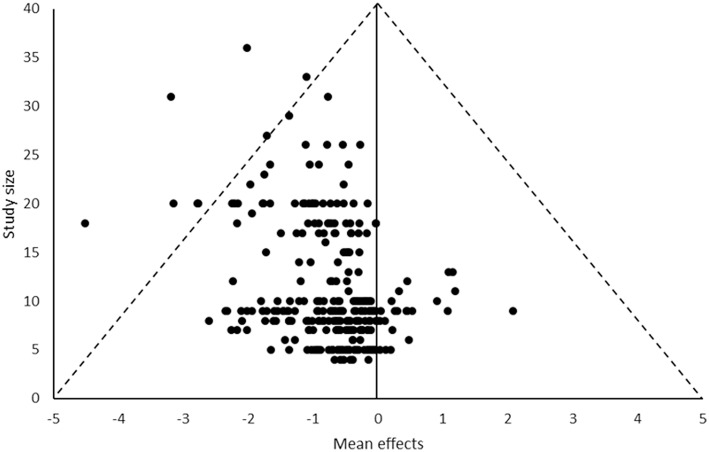
Funnel plot of all effects.

In total, 112 studies were included after duplicates removed, all of which were screened for inclusion into the analysis. There were 37 not included in the analysis due the reasons provided in the PRISMA flow diagram (Figure 1). Therefore, 75 studies (Table 2) were included, producing 922 individual effect sizes across all sub groups and outcomes. All studies were bed rest μG simulations as no astronaut studies to date included an inactive control group exposed to μG due to space agency recommended exercise programmes. There is no comparison descriptor column in Table 2 as we only considered control groups who had no intervention, treated as before and after simulated μG exposure comparisons. The most common bed rest duration was 60 days, with shortest and longest durations being seven and 120 days, respectively. The most common study design was RCT. Most of the studies scored four on the bed rest quality score, with the highest score being six, and the lowest score was two. Only three studies were assessed to have a low risk of bias. As only intervention studies' control group data were included and no actual prognostic studies were found and included, question three on the QUIPS about prognostic factors was rated as n/a for all the included studies. A rating for question 3 would have been provided had any actual prognostic studies been found and included. However, for this review, time in μG can be considered the prognostic factor and the quality of the μG simulation was critiqued in detail within the bed rest quality scores. There is some asymmetry in the funnel plot in Figure 2, suggesting potential publication bias toward studies reporting decreases in muscles, however there are studies, including smaller ones, that do report an increase. Fourty five studies specified a time period ahead of the bed rest period in which baseline measures were recorded ranging from 1 to 21 days. Of these, 11 (Greenleaf et al., 1983, 1989, 1994b; Dudley et al., 1989; Ellis et al., 1993; Ferrando et al., 1995; Portero et al., 1996; Muir et al., 2011; Lee et al., 2014; English et al., 2016; Schneider et al., 2016) stated utilizing a pre-bed rest ambulatory control period in their methods section. However, it was not clear in any of the studies what the control period involved or if there was any pre-bed rest deconditioning that was measured or adjusted for. One study, Mulder et al. (2008) measured baseline outcomes on day 4 of bed rest and acknowledges this could have led to underestimating the effect of bed rest, especially for time sensitive outcomes such as those associated with muscle. Full data tables for results per muscle are available in supplementary data tables as indicated in each results sub-section. The raw data supporting the conclusions of this manuscript will be made available by the authors, without undue reservation, to any interested parties.

**Table 2:** Characteristics of analyzed studies.

**Study ^**(analysis cross reference no.)**^**	**Design**	***n***	**Outcomes**	**Bed rest quality tool**								**TOT**	**QUIPS risk of bias tool**						
				**Days**	**1**	**2**	**3**	**4**	**5**	**6**	**7**		**1**	**2**	**3**	**4**	**5**	**6**	**Overall**
Akima et al. (2000)^1^	RCT	4	Cross-sectional area, torque, volume	20	y	?	?	y	y	?	y	4	M	L	n/a	L	H	L	H
Akima et al. (2003)^2^	RCT	6	Cross-sectional area, torque	20	y	?	?	y	y	?	y	4	M	L	n/a	L	M	L	H
Akima et al. (2005)^3^	RCT	5	Activity (EMG), volume, MVC	20	y	y	?	y	y	?	y	5	M	L	n/a	L	M	L	H
Akima et al. (2007)^4^	RCT	6	Muscle volume	20	y	?	?	y	y	?	y	4	M	L	n/a	L	M	L	H
Alkner and Tesch (2004)^5^	RCT	9	Volume, MVC, force, power, torque, activity (EMG)	90	y	?	?	y	y	?	y	4	M	L	n/a	L	M	L	H
Alkner et al. (2016)^6^	RCT	9	Activity (EMG), force	90	y	?	?	y	y	?	y	4	M	L	n/a	L	M	L	H
Arbeille et al. (2009)^7^	RCT	8	Volume	60	y	y	?	y	y	?	y	5	L	L	n/a	L	M	L	H
Bamman et al. (1997)^8^	RCT	8	MVC, activity (EMG), torque, power, work	14	y	y	?	y	y	?	y	5	L	L	n/a	L	M	L	H
Belavy et al. (2007a)^9^	RCT	10		56	n	y	?	y	?	?	y	3	L	L	n/a	L	H	L	H
Belavy et al. (2008)^10^	RCT	10	Cross-sectional area	56	?	y	?	y	?	?	y	3	L	L	n/a	L	H	L	H
Belavy et al. (2009a)^11^	RCT	10	Volume	56	?	y	?	y	?	?	y	3	L	L	n/a	L	H	L	H
Belavy et al. (2009b)^12^	RCT	10	Volume	56	?	y	?	y	?	?	y	3	L	L	n/a	L	H	L	H
Belavy et al. (2010b)^13^	RCT	9	Cross-sectional area	60	y	y	y	y	y	?	y	6	H	L	n/a	L	H	L	H
Belavy et al. (2011a)^14^	RCT	9	Cross-sectional area	60	y	y	y	y	y	?	y	6	M	L	n/a	L	M	L	H
Belavy et al. (2011b)^15^	RCT	9	Volume	90	y	y	?	y	?	?	y	4	L	L	n/a	L	H	L	H
Belavy et al. (2011c)^16^	CO	7	Cross-sectional area, muscle signal intensity	21	y	?	?	y	?	?	y	3	M	L	n/a	L	H	L	H
Belavy et al. (2013)^17^	RCT	9	Volume	60	y	y	y	y	y	?	y	6	H	L	n/a	L	H	L	H
Belavy et al. (2017)^18^	RCT	8	Muscle atrophy	56	?	y	?	y	?	?	y	3	L	L	n/a	L	H	L	H
Berg et al. (1997)^19^	RCT	7	Torque, activity (EMG), angular velocity, fiber types/size, cross-sectional area	42	y	?	?	y	y	?	y	4	M	L	n/a	L	H	L	H
Berg et al. (2007)^20^	RCT	5	MVC, cross-sectional area	35	n	?	?	y	y	?	y	3	H	L	n/a	L	H	L	H
Berry et al. (1993)^21^	CO	6	Cross-sectional area	30	y	?	?	y	?	?	y	3	H	L	n/a	L	H	L	H
Buehring et al. (2011)^22^	RCT	10	MVC, activity, jump power, jump height	56	n	y	?	y	?	?	y	3	L	L	n/a	L	H	L	H
Caiozzo et al. (2009)^23^	RCT	7	Torque, cross-sectional area	21	y	?	?	y	?	?	y	3	H	L	n/a	L	H	L	H
Cescon and Gazzoni (2010)^24^	RCT	4	Single and global motor unit conduction velocity	14	y	y	?	y	y	?	y	5	L	L	n/a	L	M	L	H
Convertino et al. (1989)^25^	B&A	8	cross-sectional area	30	y	?	?	?	?	?	y	2	H	L	n/a	L	H	L	H
de Boer et al. (2008)^26^	B&A	10	Thickness	35	n	?	?	y	y	?	y	3	M	L	n/a	L	H	L	H
Dudley et al. (1989)^27^	B&A	7	Torque	30	y	?	?	?	?	?	y	2	H	L	n/a	L	H	L	H
Duvoisin et al. (1989)^28^	TS	3	Torque velocity	30	y	?	?	?	?	?	y	2	H	L	n/a	L	H	L	H
Ellis et al. (1993)^29^	CS	5	Thickness	30	y	y	y	y	y	?	y	6	M	L	n/a	L	M	L	H
English et al. (2011)^30^	B&A	8	Torque	60	n	y	y	y	y	?	y	5	L	L	n/a	L	M	H	H
English et al. (2016)^31^	RCT	9	Torque and work	14	?	y	?	y	y	?	y	4	L	L	n/a	L	M	L	H
Ferrando et al. (1995)^32^	B&A	6	Volume	7	?	y	?	y	y	?	y	4	H	L	n/a	L	H	L	H
Ferretti et al. (2001)^33^	TS	7	Cross-sectional area, jump power	42	y	?	?	y	y	?	y	4	M	L	n/a	L	M	L	H
Fu et al. (2016)^34^	TS	8	Activity (EMG), force	45	y	y	?	y	y	?	y	5	M	L	n/a	L	M	L	H
Funato et al. (1997)^35^	TS	10	Strength, velocity	20	?	?	?	y	?	?	y	2	M	L	n/a	L	H	L	H
Gast et al. (2012)^36^	RCT	9	Jump height and power, sit-to-stand tests, sprint time, leg press (1RM)	60	y	y	y	y	y	?	y	6	L	M	n/a	L	M	L	H
Germain et al. (1995)^37^	RCT	6	Torque	28	y	?	?	y	y	?	y	4	M	L	n/a	L	H	L	H
Greenleaf et al. (1983)^38^	RCO	7	Hand grip endurance	14	?	y	?	?	?	?	y	2	H	L	n/a	L	H	L	H
Greenleaf et al. (1989)^39^	RCT	5	Work, torque	30	y	y	?	y	y	?	y	5	M	L	n/a	L	M	L	H
Greenleaf et al. (1994a)^40^	RCT	5	Volume	30	y	y	?	y	y	?	?	4	M	L	n/a	M	H	L	H
Holguin et al. (2007)^41^	RCT	11	Volume	90	Y	Y	?	Y	Y	?	Y	5	H	L	n/a	L	M	L	H
Holt et al. (2016)^42^	RCT	8	Cross-sectional area	60	y	y	y	y	y	?	y	6	L	L	n/a	L	L	L	L
Kawashima et al. (2004)^43^	B&A	10	Cross-sectional area	20	n	y	?	y	y	?	y	4	H	L	n/a	L	H	L	H
Koryak (1995a)^44^	B&A	6	MVC, force, time to peak tension, total contraction time	120	y	?	?	y	y	?	y	4	L	L	n/a	L	M	L	H
Koryak (1996)^45^	B&A	6	MVC, twitch tension, time to peak tension, total contraction time, surface action potentials	7	n	?	?	y	?	?	y	2	H	L	n/a	L	H	L	H
Koryak (1998a)^46^	B&A	6	Maximal twitch response force, strength, MVC, time-to peak tension, total contraction time	120	y	?	?	y	?	?	y	3	M	L	n/a	L	H	L	H
Koryak (1998b)^47^	RCT	4	MVC, evoked tetanic tension, maximal twitch tension, twitch time-to-peak tension, total contraction time	120	y	?	?	y	y	?	y	4	M	L	n/a	L	H	L	H
Koryak (1999)^48^	B&A	10	MVC, tension of maximal twitch, evoked tetanic tension, time to peak tension, total contraction time	120	y	?	?	y	y	?	y	4	M	L	n/a	L	M	L	H
Koryak (2002)^49^	B&A	6	Tension of maximal twitch, evoked tetanic tension, time to peak tension, total contraction time, surface action potential	7	n	?	?	y	y	?	y	3	M	L	n/a	L	M	L	H
Koryak (2010)^50^	RCT	6	MVC, twitch tension, time to peak tension, total contraction time	60	y	y	?	y	y	?	y	5	M	L	n/a	L	M	L	H
Koryak (2014)^51^	RCT	6	Volume, electromyogram	20	y	?	?	y	y	?	y	4	M	L	n/a	L	M	L	H
Kouzaki et al. (2007)^52^	RCT	6	Volume, electromyogram	20	y	?	?	y	y	?	y	4	M	L	n/a	L	M	L	H
Krainski et al. (2014)^53^	RCT	9	Volume, torque	35	y	y	y	y	y	?	y	6	L	L	n/a	L	L	L	L
LeBlanc et al. (1988)^54^	B&A	9	Cross-sectional area	35	n	y	?	y	y	?	y	4	H	L	n/a	L	H	L	H
Lee et al. (2014)^55^	RCT	24	Torque, 1RM, lean mass	60	y	y	y	y	y	?	y	6	L	L	n/a	L	L	L	L
Macias et al. (2007)^56^	RCT	15	Strength, torque	28	y	?	?	y	y	?	y	4	M	L	n/a	L	H	L	H
Miokovic et al. (2011)^57^	RCT	9	Volume	60	y	y	y	y	y	?	y	6	M	L	n/a	L	M	L	H
Miokovic et al. (2012)^58^	RCT	9	Volume	60	y	y	y	y	y	?	y	6	H	L	n/a	L	H	L	H
Miokovic et al. (2014)^59^	RCT	8	Volume	60	y	y	y	y	y	?	y	6	H	L	n/a	L	H	L	H
Muir et al. (2011)^60^	RCT	13	Strength, postural stability	90	y	?	y	y	y	?	y	5	M	H	n/a	L	M	L	H
Mulder et al. (2006)^61^	RCT	10	Cross-sectional area	56	n	y	?	y	?	?	y	3	L	L	n/a	L	H	L	H
Mulder et al. (2007)^62^	RCT	10	Torque	56	?	y	?	y	y	?	y	4	L	L	n/a	L	M	L	H
Mulder et al. (2008)^63^	RCT	8	Time to peak tension	56	?	y	?	y	y	?	y	4	L	L	n/a	L	M	L	H
Mulder et al. (2009a)^64^	RCT	9	Cross-sectional area, activity (EMG)	60	y	y	y	y	y	?	y	6	H	L	n/a	L	H	L	H
Mulder et al. (2009b)^65^	RCT	10	Knee extensor MVC	56	N	y	?	y	y	?	y	4	L	L	n/a	L	M	L	H
Narici et al. (1997)^66^	CS	8	Cross-sectional area, force	17	y	?	?	?	?	?	y	2	H	L	n/a	M	H	L	H
Pisot et al. (2008)^67^	B&A	10	Contraction time, muscle maximal displacement	35	n	y	?	y	y	?	y	4	H	L	n/a	L	H	L	H
Portero et al. (1996)^68^	B&A	12	MVC	30	y	?	?	y	?	?	y	3	H	L	n/a	L	H	L	H
Reeves et al. (2002)^69^	RCT	6	Force, resting fascicle length, fascicle length at MVC	90	y	y	?	y	y	?	y	5	M	L	n/a	L	M	L	H
Rittweger et al. (2005)^70^	RCT	9	Cross-sectional area	90	y	y	?	y	?	?	y	4	L	L	n/a	L	H	L	H
Rittweger et al. (2013)^71^	RCT	9	Cross-sectional area	90	y	y	?	y	?	?	y	4	M	L	n/a	L	M	L	H
Schneider et al. (2016)^72^	RCT	8	Torque, work, lean mass	30	y	y	?	y	y	?	y	5	H	L	n/a	L	L	L	H
Shinohara et al. (2003)^73^	RCT	6	MVC, activity EMG	20	y	y	?	y	y	?	y	5	H	L	n/a	L	H	L	H
Trappe et al. (2001)^74^	CS	8	Torque	17	y	y	?	?	?	?	y	3	H	H	n/a	L	H	L	H
Trappe et al. (2007)^75^	RCT	8	Volume force	60	y	y	?	y	y	y	y	6	L	L	n/a	L	M	L	H

*Bed rest quality scores: (1) 6 degrees head down tilt, (2) controlled diet, (3) fixed daily routine, (4) standardized bed rest phases, (5) uninterrupted bed rest, (6) restricted sunlight exposure, (7) same outcome measures for all. Quips risk of bias tool: (1) participation, (2) attrition, (3) prognostic factor measurement, (4) confounding factors, (5) statistical analysis and reporting. RCT, randomized controlled trial. CO, cross over; B&A, before and after; TS, time series; CS, cross sectional; RCO, randomized cross over*.

### Synthesized Findings

#### Muscle Volume

**Figure 3:**
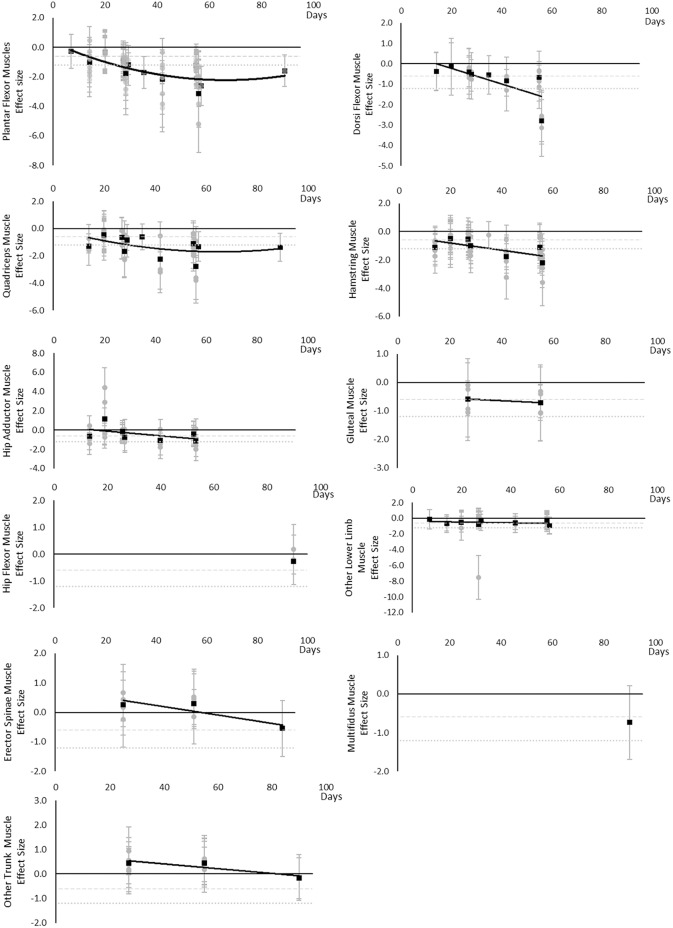
Effect size plots for muscle volume over time from individual (gray) and average (black) effect sizes at each time point, with 0.6 (dotted line) and 1.2 (dashed line) effect magnitudes and average effect trend line overlaid.

All muscle volumes decreased over time. Moderate effects were becoming apparent by 14 days and large by 28 days. Very little data were available for Hip Flexor, Gluteal, Multifidus, and Erector Spinae muscles, where a moderate or greater effect was never reached for Hip Flexors and Erector Spinae muscles and only a moderate effect was apparent by 27 and 90 days for Gluteal and Multifidus muscles, respectively. Other lower limb muscles that included Gracilis, Sartorius, Piriformis, Obturators, and Pectineus muscles, reached a moderate effect by 14 days. Other trunk muscles that included Levator Scapulae, Longus Colli, Sternocleidomastoid, and Scalene muscles never reached a moderate effect. The breakdown of individual volume effects per muscle is available in Supplementary Table 1 and associated summary plots in Figure 3.

#### Muscle Cross Sectional Area

**Figure 4:**
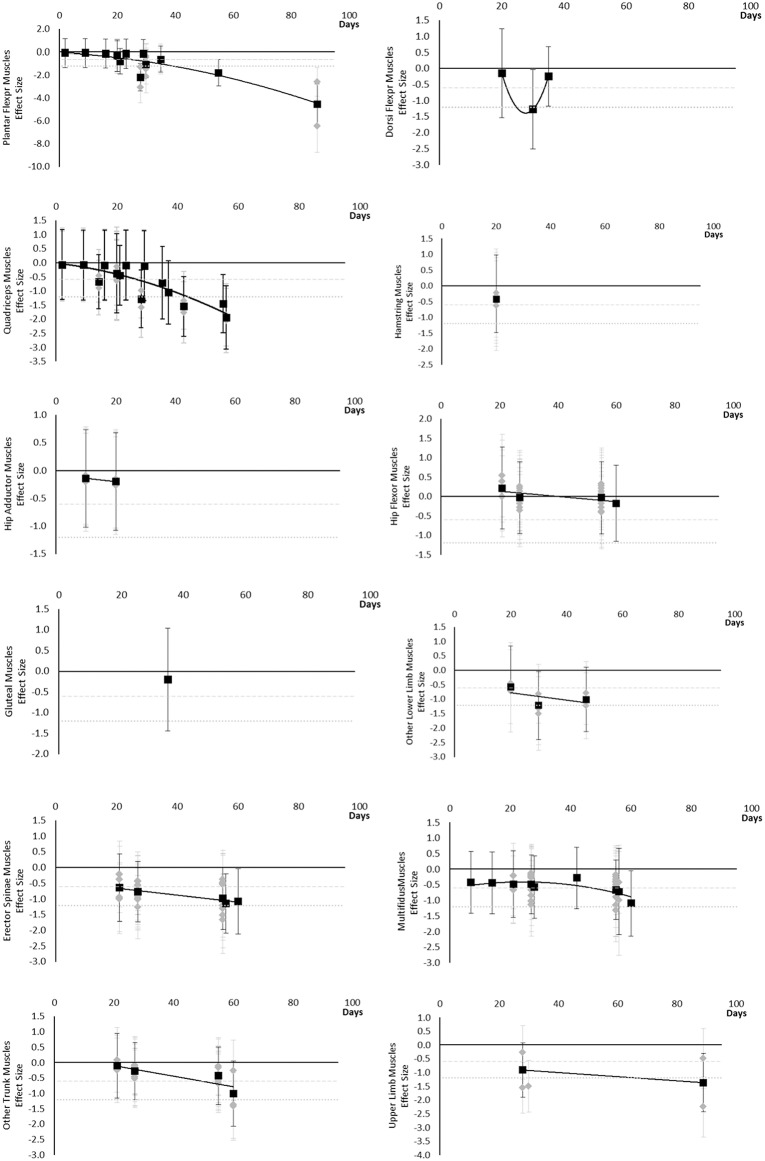
Effect size plots for muscle cross sectional area over time from individual (gray) and average (black) effect sizes at each time point, with 0.6 (dotted line) and 1.2 (dashed line) effect magnitudes and average effect trend line overlaid.

All muscle cross sectional areas decreased over time. Moderate effects were apparent by 14 days and large by 28 days. The same effect time points were found for other lower limb muscles that included Gracilis and Sartorius muscles and total thigh and calf cross sectional area. Very little data were available for Hip Flexor, Gluteal, Hamstring and Hip Adductor muscles where a moderate or greater effect was never reached. Multifidus and other trunk muscles, including Quadratus Lumborum and combined Multifidus and Erector Spinae cross sectional area, only reached a large effect by 60 days. Upper limb muscle outcomes consisted of forearm muscle cross sectional area which only reached a large effect after 89 days. The breakdown of individual cross sectional area effects per muscle are available in Supplementary Table 2 and associated summary plots in Figure 4. The polynomial trend for Dorsi Flexor muscles appeared to be unreliable.

#### Torques and Strength

**Figure 5:**
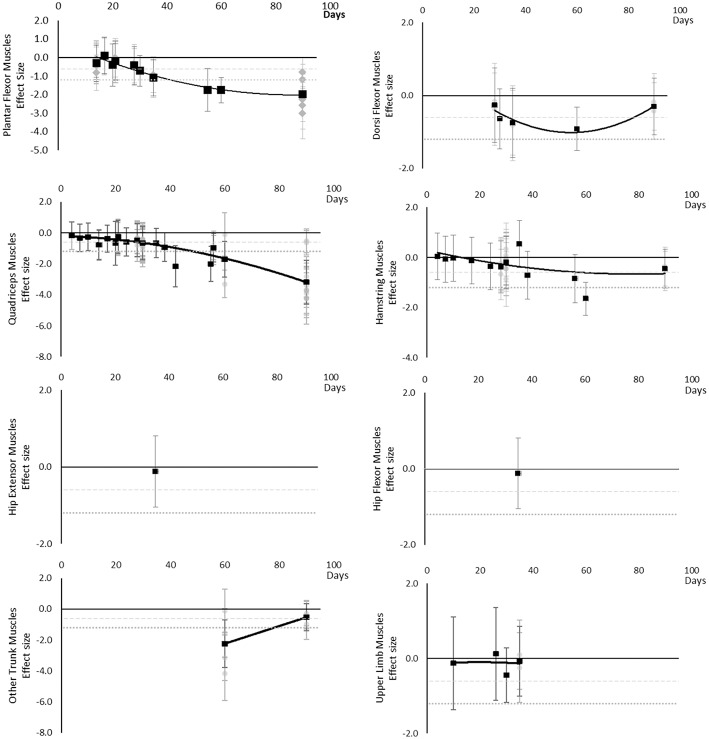
Effect size plots for torques and strength over time from individual (gray) and average (black) effect sizes at each time point, with 0.6 (dotted line) and 1.2 (dashed line) effect magnitudes and average effect trend line overlaid.

Torques and strengths decreased over time. Moderate effects became apparent by 14 days for Quadriceps muscles only. Additional moderate effects became apparently by 30 days and large effects by 35 days. Dorsi Flexor, Hamstring, Hip Extensor, Hip Flexor, other trunk, and other upper limb muscles never reached a large effect. Other trunk muscles included trunk flexors and extensors tested in combination within functional movements. Upper limb muscles included elbow flexor and extensor muscles and shoulder abductor and adductor muscles. The breakdown of individual torques and strength effects per muscle is available in Supplementary Table 3 and associated summary plots in Figure 5. The polynomial trend for Dorsi Flexor muscles appeared to be unreliable after 60 days.

#### Contractile Work Capacity

**Figure 6:**
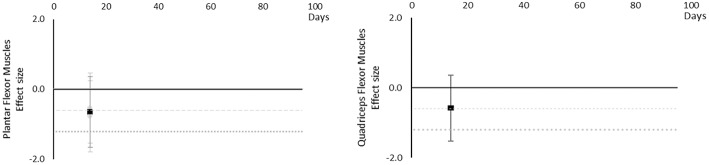
Effect size plots for contractile work capacity over time from individual (gray) and average (black) effect sizes at each time point, with 0.6 (dotted line) and 1.2 (dashed line) effect magnitudes and average effect trend line overlaid.

Although there are very little available data for contractile work capacity it appears to decrease over time. Moderate effects became apparent by 14 days in Plantar Flexor and Quadriceps muscles. However, this is based on only one study for each muscle at 14 days. The breakdown of individual contractile work capacity effects per muscle is available in Supplementary Table 4 and associated summary plots in Figure 6. Data were limited for all muscles.

#### Muscle Thickness

**Figure 7:**
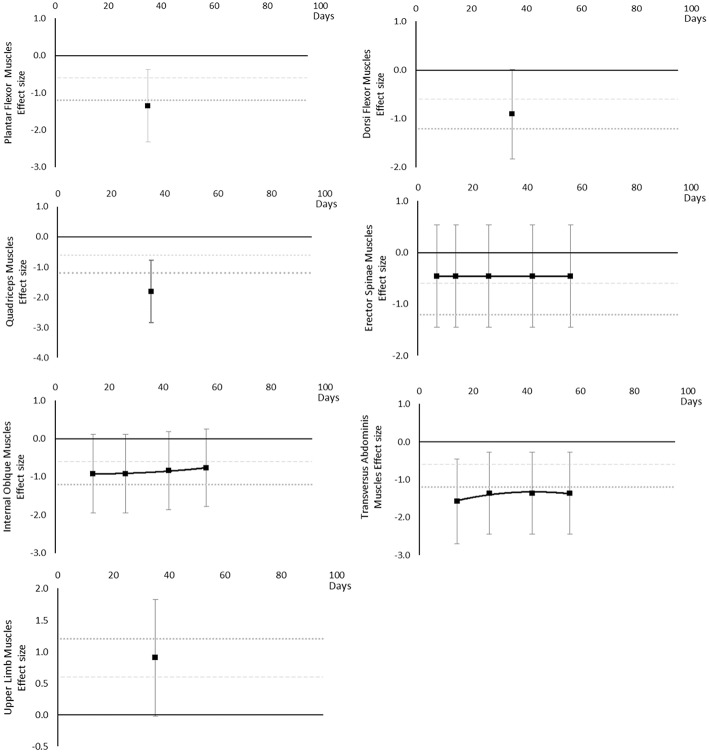
Effect size plots for muscle thickness over time from individual (gray) and average (black) effect sizes at each time point, with 0.6 (dotted line) and 1.2 (dashed line) effect magnitudes and average effect trend line overlaid.

Muscle thickness decreased over time. Moderate and large effects became apparent by 14 days. There were very little data for Plantar Flexor, Dorsi Flexor and Quadriceps muscles, showing Dorsi Flexor muscles reached a moderate effect by 35 days and only Plantar Flexor and Quadriceps muscles reached a large effect by 35 days. Internal Oblique muscle reached a moderate effect at 14 days. Erector Spinae muscle was similar to Internal Oblique muscle, but only reached a borderline moderate effect within the available data. Upper limb muscles data only included Biceps Brachii muscle thickness, reaching a moderate effect by 35 days. The breakdown of individual muscle thickness effects per muscle is available in Supplementary Table 5 and associated summary plots in Figure 7.

#### Peak Power

**Figure 8:**
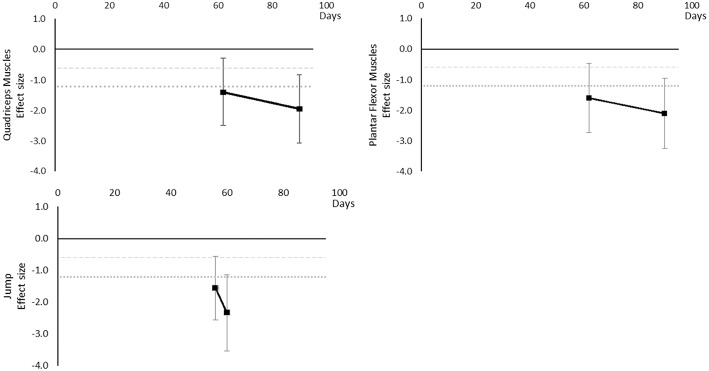
Effect size plots for peak power over time from individual (gray) and average (black) effect sizes at each time point, with 0.6 (dotted line) and 1.2 (dashed line) effect magnitudes and average effect trend line overlaid.

Peak power decreased over time. Large effects became apparent by 56 days for jump power and 62 days for Plantar Flexor and Quadriceps muscles. There was insufficient data to determine a time point for when any moderate effects were reached. The breakdown of individual peak power effects per outcome is available in Supplementary Table 6 and associated summary plots in Figure 8.

#### Muscle Activity

**Figure 9:**
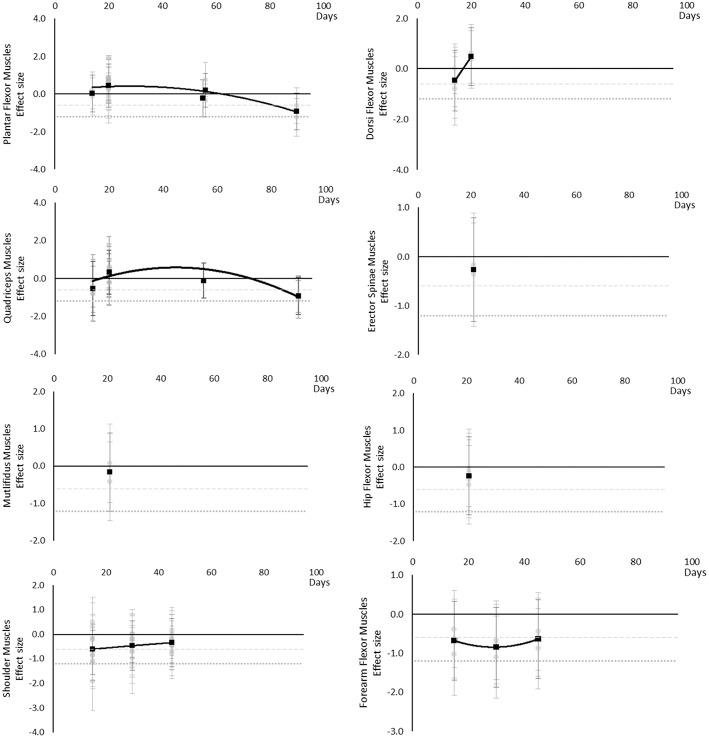
Effect size plots for EMG muscle activity over time from individual (gray) and average (black) effect sizes at each time point, with 0.6 (dotted line) and 1.2 (dashed line) effect magnitudes and average effect trend line overlaid.

Muscle activity (via electromyography) generally decreased over time, however a transient increase was seen in Plantar Flexor, Dorsi Flexor and Quadriceps muscles and only at 20 days. In Plantar Flexor and Quadriceps muscles, muscle activity decreased again after 20 days, there were no data for Dorsi Flexor muscles beyond 20 days to establish a post 20 day trend. Moderate effects were apparent in upper limb muscle groups by 15 days but not until 90 days for Dorsi and Plantar Flexor muscles which were the only muscles with data at the 90 day point. The breakdown of individual activity effects per muscle is available in Supplementary Table 7 and associated summary plots in Figure 9.

#### Maximal Voluntary Contraction During One Repetition Maximum

**Figure 10:**
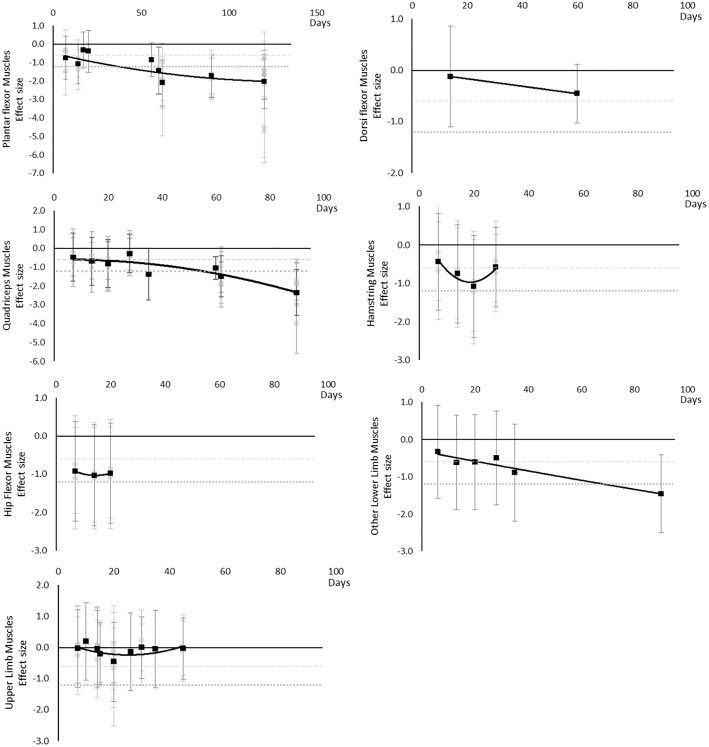
Effect size plots for MVC during one rep max over time from individual (gray) and average (black) effect sizes at each time point, with 0.6 (dotted line) and 1.2 (dashed line) effect magnitudes and average effect trend line overlaid.

Maximal voluntary contraction during one repetition maximum decreased over time except for other upper limb outcomes that remained mostly unchanged as far as data were available up to 45 days. Moderate effects became apparent by 7 days and large effects by 35 days. Other lower limb outcomes that included maximal isometric force during supine squat, hip extensor force and legs total work never reached a large effect, but had no data available beyond 35 days. The breakdown of individual MVC during one repetition maximum effects per muscle is available in Supplementary Table 8 and associated summary plots in Figure 10. The polynomial trend for Hamstring muscles appeared to be unsafe after 20 days.

#### Power

**Figure 11:**
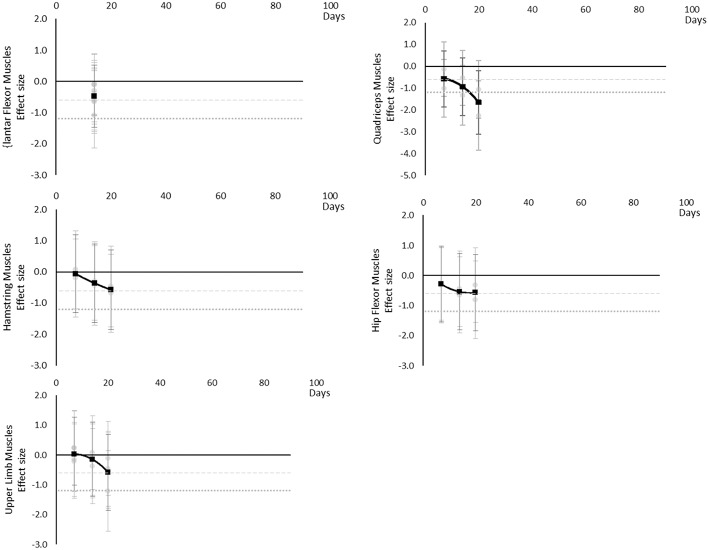
Effect size plots for power over time from individual (gray) and average (black) effect sizes at each time point, with 0.6 (dotted line) and 1.2 (dashed line) effect magnitudes and average effect trend line overlaid.

Power decreased over time. Moderate effects became apparent by 7 days and large effects by 20 days, although these were only seen in the Quadriceps muscle data. Hamstring, Hip Flexor, and upper limb muscles that included elbow flexors and extensors reached moderate effects by 20 days. Plantar Flexors never reached a moderate effect but data were only available at 14 days. Other trunk muscles included trunk flexors and extensors tested in combination within functional movements. The breakdown of individual power effects per muscle is available in Supplementary Table 9 and associated summary plots in Figure 11.

#### Performance Based

**Figure 12:**
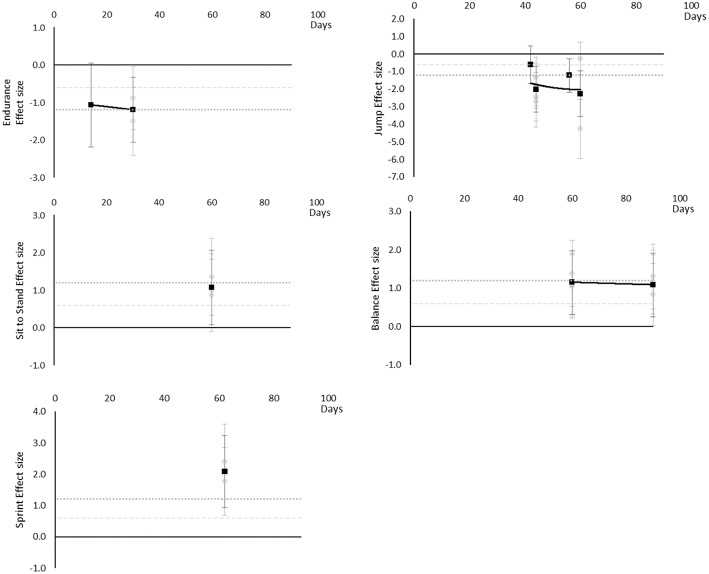
Effect size plots for performance based over time from individual (gray) and average (black) effect sizes at each time point, with 0.6 (dotted line) and 1.2 (dashed line) effect magnitudes and average effect trend line overlaid.

Performance based outcomes all worsened over time, as although sit to stand, balance, and sprint time outcomes all had positive effects, this was considered a worsening effect within these measures. Endurance reached a large effect by 14 days, jumping a moderate effect at 42 days and large by 44 days, sit to stand and balance reached large effects by 60 days and sprint time by 62 days. Data for most outcomes were only available for one time point and so trends over time for individual outcomes are not able to be determined. The breakdown of individual performance based effects per outcome is available in Supplementary Table 10 and associated summary plots in Figure 12. It should be noted that while these outcomes are grouped as being performance based for this review, they may differ and each individual measure should be considered on its own merit.

## Discussion

### Summary of Main Findings

The main finding of the review was that muscle cross-sectional area, volume, shape, size, activity, power, performance, torque, and force-based outcomes, at either regional or global level, all decline over time, based on the current evidence base. Moderate effects became apparent in the following order: power and MVC during one repetition maximum (7 days), followed by volume, cross sectional area, torques and strengths, contractile work capacity, thickness and endurance (14 days), then muscle activity (15 days). Large effects became apparent in the following order: volume, cross sectional area (28 days) torques and strengths, thickness (35 days), and peak power (56 days). No large effects were found for muscle activity. There were limited data for contractile work capacity and no large effects were apparent. In general, lower limb and trunk muscles appeared to decline more rapidly than upper limb muscles. Locomotion muscles such as Plantar Flexor and Quadriceps muscles also generally appeared to decline more rapidly than other muscles groups and with larger effect sizes.

### Findings Within Context of Human Space Mission Profiles

**Figure 13:**
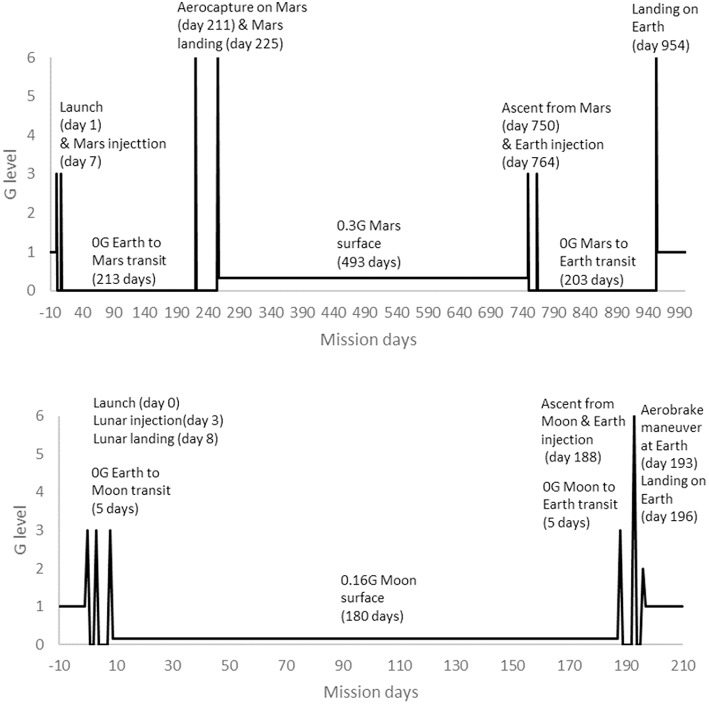
Mission profiles for 180 day surface stay Lunar mission (Bottom) and 493 day surface stay Mars mission (Top), adapted from HUMEX (Horneck et al., 2003, 2006).

Human spaceflight missions differ in duration, so results have to be placed into the context of mission profiles and operationally important considerations. Operationally, performance-related measures such as power, MVC, torques and strengths are considered most critical. In terms of mission profiles, typical ISS missions involve approximately 180 days in μG (Bryant et al., 2017). The provision of time for exercise CM is mandated for these missions and developments have led to improved efficacy over the lifetime of ISS (Trappe et al., 2009; Ploutz-Snyder, 2013; Hackney et al., 2015). Assuming that the rate of change during bed rest is reasonably transferable to that experienced in μG, the results of this systematic review suggest that large effects would be apparent within a 180 day ISS mission if no exercise CM were employed. That ISS astronauts are able to complete missions without problems from muscle deterioration and successfully return to Earth may provide some level of evidence with which to judge current countermeasures as effective. However, the focus of this review is exploration beyond Low Earth Orbit. Lunar and Martian (exploration) mission profiles were defined in the HUMEX study (Horneck et al., 2006) that modeled exploration mission durations including transit times in μG and planetary stay times in low (<1 G) gravity (Figure 13). HUMEX defined three scenarios, including a Lunar mission with a 180 day surface stay (Horneck et al., 2003) and two Mars missions with either a 30 or 400 day surface stay (Horneck et al., 2006). In HUMEX, inter planetary transit time in μG was 5 days for Lunar missions and 203–213 days for Mars.

#### Mars

It is clear from the findings of this review that changes in muscle outcomes, including performance related measures, with large effects would be observed if no CM were performed during a 200+ day transit to Mars. A risk assessment (Gernand, 2004) has highlighted that decreased muscle mass, strength, and endurance is likely to lead to inability to complete mission critical tasks such as exiting a spacecraft on landing, performing strenuous extra vehicular activity and being functional during increased Gz loading on non-Earth planetary surfaces where a landing support and rehabilitation team may not be available. Therefore, effective CM to prevent muscle deterioration are likely going to be required for Mars missions unless absolute strength mission requirements can be reduced or eliminated, to mitigate risks of crews being unable to perform mission critical tasks and continue to function safely on arrival at Mars. However, based on the occurrence of large effect sizes in the present results only after 28–35 days, exercise CM “holidays” might be considered during Mars transits/orbits to save resources if agencies were confident that moderate changes in muscle performance could be reversed using in-flight exercise equipment and prescriptions.

#### Moon

The results of this review suggest that exercise CM might not be required during a 5 day Lunar transit period, as moderate effects on muscle are not likely to be apparent until 7 days. The initial changes in power and MVC might not be functionally limiting enough to risk mission success, compared to muscle size, strength, and endurance effects that do not reach a moderate size until 14 days. Therefore, further investigation of any effects within the expected Earth-Lunar transit period, considered against minimal clinically worthwhile and mission critical magnitude changes, may be useful to confirm this finding. As a Lunar landing may occur at 8 days in the HUMEX models, the pre-flight strength of crew and the absolute strength and functional requirements of Lunar landing activities would need to be considered when deciding whether or not employ exercise CM prior to attempting a landing. While not employing exercise CM might be considered for the Earth-Lunar transit period, a recent systematic review of biomechanical responses to reduced gravity (Richter et al., 2017) showed that exercise CM would likely be needed during stays on the planetary surfaces of both Moon (0.16 g) and Mars (0.38 g). As time in both μG and low gravity accumulates over the entire mission duration (196-d in total for the HUMEX model), exercise CM are also likely to be needed during the return to Earth transit. However, not using exercise CM on the return transit might be considered if key muscle outcomes could be maintained at, or restored to, pre-mission levels by the end of a Lunar surface stay. Based on the occurrence of large effect sizes in the present results, in an off-nominal situation, such as an emergency, a longer period, possibly up to around 30 days, without exercise CM might be considered if the risks of moderate-large effects can be managed in some other way, for example, knowing support and a full rehabilitation programme are available at the destination arrival site. As with Mars missions, for long Lunar orbital missions with extended periods in μG, an exercise CM “holiday” of the same duration might be considered if agencies were confident that moderate changes in muscle performance could be reversed in-flight.

#### Individuals More Susceptible to μG Induced Muscle Changes

An individual with a relatively lower muscle outcome measure may be more susceptible to experiencing a negative functional impact of negative changes in these outcomes compared to someone with greater initial measures. It is expected that most missions will require an absolute (minimal) level of strength to achieve mission critical tasks such as donning/doffing and standing up/moving whilst wearing a space suit in low gravity, hatch opening, and pulling/dragging a fellow crew member wearing a space suit during an emergency. The absolute level is defined as the precise required strength outcome in raw units to achieve a task, as opposed to considering relative changes with effect size or percentage changes. A relative (%) reduction in strength will make all tasks with an absolute strength requirement more challenging for all individuals, but the biggest impact will be felt by those who have a lower initial level of absolute strength. For example, a strong individual might be able to lose 30% of their pre-flight strength and still comfortably achieve a mission critical task (and also still be stronger than a weaker individual was prior to flight), whereas a weaker individual might already be close to their physical limit during this task without any deconditioning. Operationally, having an estimate of the most rapid possible rate of change in muscle outcomes may be useful in the case of a crew member with low pre-flight absolute strength, or an individual highly susceptible to μG adaptation. In the present study, the most extreme negative value within the confidence interval for each outcome provides an estimate of the most extreme worst likely true value that might be encountered with exposure to μG. Based on this estimation, the results of this analysis suggest that the change experienced by an individual astronaut might reach a large effect size in some muscles within a 7 day lunar transit period for volume, cross sectional area, contractive work capacity, thickness, power, and MVC. However, the confidence intervals are wide due to the small sample sizes across the current evidence base, so this estimate should be treated with caution as it may be exaggerated. Individual effects are difficult to determine in a transferable way to the true population from the data currently available or from individual case studies. Ideally, a population selected for their increased susceptible to unloading/μG-induced muscular adaptation should be studied in a long-duration μG analog to produce a representable average effect that could be transferred to the true population with more reasonable confidence. Until such data are available, estimating the maximum rate of decline in an individual in response to μG exposure of such a duration will remain difficult. In addition, consideration would also be needed should an individual be selected to perform some tasks in a mission that are not considered mission critical, but are essential to other mission goals. It may be that checking for susceptibility to outcomes that are linked more strongly to mission success is checked and made part of astronaut eligibility screening, it could also be any more susceptible mission critical individuals undergo more rigorous preflight and inflight training protocols or consider use of other more removed countermeasures beyond the scope of this review.

Exercise countermeasure development may want to consider focusing on those which might best address the more susceptible outcome changes in this review, volume, cross sectional area, contractive work capacity, thickness, power, and MVC while also ensuring any proposed exercises are tailored to tasks considered critical, such as donning/doffing and standing up/moving whilst wearing a space suit in low gravity, hatch opening, and pulling/dragging a fellow crew member wearing a space suit. The impact of any chosen exercise types on future spacecraft exercise hardware would also need further consideration. Future research should consider identifying exercise countermeasures that would best address the more susceptible outcomes and be feasible with any technical constraints of new space vehicles planned for use within Moon and Mars missions.

#### Countermeasure Requirement

As CM are likely to be needed on the return trip from both Moon and on the journeys to and from Mars, such CM will need developing. Countermeasure devices should support lower limb and trunk muscle exercise as these decline earlier than other body regions and are essential for locomotion and for spinal function on return to G loading (Bamman, 1996; Pavy-Le Traon et al., 2007; Evetts et al., 2014; Stokes et al., 2016; Winnard et al., 2017). Based on the results of the present study, if exercise CM are used during very short missions/transits (e.g., up to 7 days), devices should support exercise that maintains power and maximal force production, as moderate effects appeared early in these performance outcomes. Up to around 15 days, exercise CM might need only to prevent moderate size effects in muscle. Consideration could be made around if lower intensity exercise, or potentially a break in countermeasures would be safe. However, once μG exposure duration reaches around 30 days and above, large effects in muscle will likely need to be managed and this would likely require devices/prescriptions optimized within the constraints of the vehicle/habitat.

This pattern fits current European Space Agency (ESA) ISS Long Duration Mission (LDM) exercise prescriptions (Petersen et al., 2016) that include an initial 20-day familiarization phase to allow crew to adjust to exercise in μG and minimize injury risk, in which exercise intensity is moderate compared to pre-flight maximum capacity. However, as there is currently no systematic measurement of muscle performance in-flight, the impact of this period of lower intensity exercise on overall changes in muscle during an LDM is unknown. It is also unclear if crew members may have had better results at the end of a mission had they begun exercising more intensely earlier in the mission. Following the 20-day familiarization period, exercise prescriptions are increased in intensity to 80%+ maximal capacity. In the final 15–30 days of a long duration mission (>49 days) intensity is kept high, but focus on resistance and running exercises. In flight resistance exercise prescriptions for European astronauts also focus on lower limb muscles (squats, heel raises, deadlifts) ESA has found are most susceptible to μG induced changes from (non-systematic) measures that have been taken (Petersen et al., 2016). Similar exercise prescriptions, focusing on lower limb muscles and maintaining outcomes already highlighted in this review, might form a good basis for initial planning for any exercises required for Lunar and Mars missions. Additionally, research on preventing deconditioning of older adults might also be useful as preventing loss of power in functional lower limb muscles is important in this population and simple loading exercises have shown helpful in this context (Byrne et al., 2016). It should also be noted, however, that a systematic review of in-flight CM for maintaining spinal health in μG found that, while resistance based exercises helped prevent muscle changes, they did not help with non-muscle outcomes such as spinal morphology (Winnard et al., 2017). Moreover, a number of other physiological systems/organs also adapt to μG, including bone and aerobic capacity, but the efficacy of resistance exercise during gravitational unloading on them is unknown as systematic reviews similar to the present study have yet to be performed. Therefore, while the recommendations of this review are expected to help plan CM for *muscle* changes, additional holistic consideration of other physiological systems will likely be required. Finally, any CM development for exploration missions will also have to consider constraints of space vehicles that will be used, such as available physical space, limited number of devices that can be included in the space craft, consumables, generation of heat, carbon dioxide, and vibration, which are likely to be more restricted than the ISS (Hackney et al., 2015). Before any pause in exercise countermeasures could be taken, the results of this review would need to be validated in microgravity and ideally actual astronauts through experimental studies. No such published studies of astronauts not performing exercise to document muscle changes over the time frames considered in this review was found. Space agencies and researchers would also need to consider the ethical implications and acceptability of any such study.

### Completeness and Quality of Current Evidence

There were missing and limited data across all the outcome measure subgroups, and gaps in the evidence base were clearly shown in the results tables. There was a lack of standardized time points at which measures were recorded, even across studies reporting the same outcome measures. Limited data were found repeatedly for Gluteal and Hip Flexor muscles across several outcome measure subgroups. Data were lacking for contractile work capacity, muscle thickness, and peak power outcome measures where further research is recommended to validate the trends seen over time in the current evidence base. No patient reported outcome measures have been reported across the bed rest studies, meaning it is unclear how relevant the measures are to patients (in this case astronauts) (Dawson et al., 2010; Nelson et al., 2015). In addition, only seven out of the 75 analyzed studies considered functional performance based outcomes that are more likely to be directly relevant to astronauts. While strong efforts on behalf of space agencies to standardize bed rest studies has occurred including listing required surrogate measures (Sunblad et al., 2014), patient reported outcomes such as their ability to perform a task felt of value to them, remain missing on the whole. It is recommended that the scientific and space medical operations communities agree on set times points at which outcome measures should be tested to enable easier comparisons across studies and for overall trends to be more easily identifiable. While ESA requires agency bed rest studies to be performed to set standards, it might be beneficial to consider running a specific initiative in the wider Aerospace Medicine field to establish core outcome sets relevant to space medicine operations that should then be used in all associated research. This could be based on recommending use of standard space agency developed tests such as functional and Field Test parameters developed by NASA and Russia The Core Outcome Measures in Effectiveness Trials (COMET) is an example initiative that facilitates development and application of core outcome sets and research has been published on how to reach consensus using such an approach (Prinsen et al., 2014). It is also recommended that patient reported outcome measures, and increased reporting of functional performance based outcome measures, be included in both future research and space medical operations to ensure that outcome measures are assessing phenomena that are relevant to astronauts. This recommendation echoes a recent European Space Agency topical team report that also found patient reported outcome measures not being used in space medicine research and operations (Stokes et al., 2016). The report recommended the use of such outcomes and suggested potential for development of new such outcome measures specifically for space medicine with operational space medicine input to ensure relevance across research and clinical settings. It would be of further benefit if clinically worthwhile, or concerning, changes were defined for key outcome measures, so that results can be placed into a clinically meaningful context. Reporting results based on clinically meaningful raw changes would likely be more informative to operational decisions compared to the more mechanistic null hypothesis tests, effect size or percentage change measures currently used. The high risk of bias and lack of core outcome measure sets means that the conclusions reached by this review should be treated with some caution. A bed rest study could be performed to confirm the findings of this review. If performed, the study would ideally be a randomized controlled trial comparing inactive bed rest with controls not performing bed rest but controlled for all potential confounding factors. For example, exercise and any other types of muscle interventions would need to be strictly controlled for the period of the study. The bed rest element would ideally comply with all aspects of the AMSRG bed rest quality tool to improve transferability of results to astronauts (Winnard and Nasser, 2017b). Finally, all modifiable risk of bias elements would need controlling and a risk of bias tool for randomized controlled trials, such as provided by Cochrane (Higgins et al., 2011), could be used as a guide to check what elements need to be controlled to minimize bias risks.

Most of the studies scored four on the bed rest tool, with no studies scoring a full seven points, although 13 studies scored six. The reasons for marking studies down was mostly due it being unclear if criteria had been met rather than clearly failing a point. The most common unclear criteria was related to restricted sunlight exposure followed by ensuring a fixed daily routine. The high risk of bias results were most commonly caused by not clearly showing how confounding factors were managed and providing adequate description of participation. The participation domain considers participant eligibility criteria, source of participants, baseline descriptions, description of sampling frame and recruitment, description of period and place of recruitment, and inclusion/exclusion criteria (Hayden et al., 2013). The sunlight exposure criteria has more impact on bone outcomes (Holick, 2004) due to its role in vitamin D levels within human bone homeostasis (Tarver, 2013) so might not be a large concern for the muscle outcomes presented in this review. However, it is recommended that future bed rest protocol information be clear on all the criteria assessed on the bed rest quality tool and especially on the fixed daily routine and restricted sunlight points, while also ensuring that information is provided about control of confounding factors to help reduce risk of bias and participation considerations. In addition, studies that assess time sensitive outcomes, such as muscle (in which the results of this review show effects of deconditioning can occur by 7 days), should report any potential for pre-bed rest deconditioning during familiarization and baseline measure periods and any attempts to control for this. There is potential that participants who are admitted to bed rest facilities several days in advance for control measures could decondition within this period. Some studies state including an ambulatory control period, but none report details of what this involved or if there was potential for pre-bed rest deconditioning to influence results.

There was some asymmetry in the funnel plot showing potential publication bias toward studies reporting a decrease in muscle outcomes. However, there were studies present on the increasing side of the plot, so the risk is not likely to be high. In addition, it is expected that many of the muscle outcomes would decrease during a period of inactivity such as bed rest, therefore, it not surprising most studies reported decreases. Therefore, while it appears a risk of reporting bias may exist, the presence of some studies reporting increases and the expected pattern of more decreases being reporting suggest this finding should be treated with caution and the potential risk is likely to be low.

### Limitations

This review only considered muscle outcomes. Spaceflight is known to affect many more human physiological systems including bone, cardiovascular and vestibular (Pavy-Le Traon et al., 2007). These results alone, therefore, only provide a muscle based perspective. As typical meta-analysis statistics assume two independent groups (Higgins and Green, 2011), a more basic effect size analysis without these assumptions had to be used due to only considering changes over time in the control group of each study. Therefore, some caution should be taken as the mean effect sizes are not weighted and heterogeneity scores are not available. However, as most studies had small sample sizes, a weighted result is not expected to produce largely different results. Additionally, the findings of this review appear to match actual spaceflight findings and patterns, such as the European Space Agency exercise prescription for long duration missions that performs lower intensity exercises for the first 20 days. While actual measures are not taken during flight, the 20 days has so far not resulted in any mission critical functional decline (Petersen et al., 2016). The 20 day period would fit with the findings of this review that only moderate effects would be expected before 28 days and gives some partial validation, from actual astronaut data, to the findings of this review. The review is also broad and, in places, the variation around the outcomes appears large suggesting heterogeneity of data may be high, although the large intervals could also be due to the small sample sizes that were a common feature of the included bed rest studies. Due to the broad data set that summarizes the entire muscle evidence base, additional data on pre-bed rest fitness of participants was not extracted for analysis. While studies were selected that had healthy adults undergoing spaceflight simulation bed rest, individual physical condition was not considered beyond this. Therefore, there may be some limitations to the transferability of astronauts who undergo training with space agencies prior to missions. However, a broad summary of the entire current evidence base with basic effect size analysis was the best way to try to address the overarching research questions, look for high level trends, and present a summary of the current state of the complete evidence base.

### Conclusions

The results of this review suggest that moderate effects on a range of muscle function parameters may occur within 7–14 days of unloading, with large effects within 35 days. Combined with identification of muscle performance requirements for future exploration mission tasks, these data, may support the design of CM programmes to optimize their efficient use without compromising crew safety and mission success. However, the data suggests CM are likely to still be needed for longer transit/orbital periods of 14–28+ days, such as a prolonged Lunar orbit, deep space exploration, or a Mars mission, as moderate effects occur between 7–14 days and large effects by 28 days for most muscle outcomes. However, if large effect sizes occur only after 28–35 days, to save resources, space agencies might consider short missions without exercise CM, or fixed periods of abstinence during longer μG exposures, if they could be confident that moderate changes in muscle performance could be reversed in-flight. Finally, several research gaps are highlighted for future bed rest studies in which standardized time points for measurements should be used and clear information provided on sunlight exposure control, fixed daily routine, and control of any confounding factors.

## Analyzed Study List

^1^Akima et al., 2000; ^2^Akima et al., 2003; ^3^Akima et al., 2005; ^4^Akima et al., 2007; ^5^Alkner and Tesch, 2004; ^6^Alkner et al., 2016; ^7^Arbeille et al., 2009; ^8^Bamman et al., 1997; ^9^Belavy et al., 2007a; ^10^Belavy et al., 2008; ^11^Belavy et al., 2009a; ^12^Belavy et al., 2009b; ^13^Belavy et al., 2010a; ^14^Belavy et al., 2011a; ^15^Belavy et al., 2011c; ^16^Belavy et al., 2011b; ^17^Belavy et al., 2013; ^18^Belavy et al., 2017; ^19^Berg et al., 1997; ^20^Berg et al., 2007; ^21^Berry et al., 1993; ^22^Buehring et al., 2011; ^23^Caiozzo et al., 2009; ^24^Cescon and Gazzoni, 2010; ^25^Convertino et al., 1989; ^26^de Boer et al., 2008; ^27^Dudley et al., 1989; ^28^Duvoisin et al., 1989; ^29^Ellis et al., 1993; ^30^English et al., 2011; ^31^English et al., 2016; ^32^Ferrando et al., 1995; ^33^Ferretti et al., 2001; ^34^Fu et al., 2016; ^35^Funato et al., 1997; ^36^Gast et al., 2012; ^37^Germain et al., 1995; ^38^Greenleaf et al., 1983; ^39^Greenleaf et al., 1989; ^40^Greenleaf et al., 1994b; ^41^Holguin et al., 2007; ^42^Holt et al., 2016; ^43^Kawashima et al., 2004; ^44^Koryak, 1995a; ^45^Koryak, 1996; ^46^Koryak, 1998a; ^47^Koryak, 1998b; ^48^Koryak, 1999; ^49^Koryak, 2002; ^50^Koryak, 2010; ^51^Koryak, 2014; ^52^Kouzaki et al., 2007; ^53^Krainski et al., 2014; ^54^LeBlanc et al., 1988; ^55^Lee et al., 2014; ^56^Macias et al., 2007; ^57^Miokovic et al., 2011; ^58^Miokovic et al., 2012; ^59^Miokovic et al., 2014; ^60^Muir et al., 2011; ^61^Mulder et al., 2006; ^62^Mulder et al., 2007; ^63^Mulder et al., 2008; ^64^Mulder et al., 2009bb; ^65^Mulder et al., 2009a; ^66^Narici et al., 1997; ^67^Pisot et al., 2008; ^68^Portero et al., 1996; ^69^Reeves et al., 2002; ^70^Rittweger et al., 2005; ^71^Rittweger et al., 2013; ^72^Schneider et al., 2016; ^73^Shinohara et al., 2003; ^74^Trappe et al., 2001; ^75^Trappe et al., 2007.

## Not Analyzed Study List

^1^Belavy et al., 2007b; ^2^Belavy et al., 2010b; ^3^Belavy et al., 2012; ^4^Biolo et al., 2008; ^5^Cavanagh et al., 2016; ^6^Shenkman et al., 1997; ^7^Amorim et al., 2006; ^8^Rittweger and Felsenberg, 2009; ^9^Koriak Iu, 2010; ^10^Koriak Iu, 2012; ^11^Koriak Iu, 2013; ^12^Koryak, 1994; ^13^Bamman and Caruso, 2000; ^14^Bamman, 1996; ^15^Felsenberg et al., 2009; ^16^Ferretti, 1997; ^17^Greenleaf et al., 1994a; ^18^Grogor'eva and Kozlovskaia, 1987; ^19^Guo et al., 2001; ^20^Hargens et al., 2003; ^21^Judith Hayes et al., 1992; ^22^Ito et al., 1994; ^23^Jaweed et al., 1995; ^24^Koryak, 1995b; ^25^Kozlovskaia et al., 1984; ^26^LeBlanc et al., 1992; ^27^LeBlanc et al., 1997; ^28^Liu et al., 2003; ^29^Macias et al., 2008; ^30^Meuche et al., 2006; ^31^Meuche et al., 2005; ^32^Milesi et al., 1997; ^33^Miyoshi et al., 2001; ^34^Moriggi et al., 2010; ^35^Mulder et al., 2011; ^36^Netreba et al., 2004; ^37^Scott et al., 2017.

## Author Contributions

AW: initial concept ideas, protocol planning and drafting, search screening, analyzing, and drafting all manuscript versions. JS: methods advice, protocol drafting, and approving final draft. NW: data extraction, analysis, and drafting final version. MV: protocol planning, search screening, data analysis, drafting text, and checking final version. NC: protocol planning, search screening, methods advice, manuscript drafting, and approving final version.

### Conflict of Interest Statement

The authors declare that the research was conducted in the absence of any commercial or financial relationships that could be construed as a potential conflict of interest.
