# Supplementary material for: Effect of Time on Human Muscle Outcomes During Simulated Microgravity Exposure Without Countermeasures—Systematic Review
Source: Front Physiol. 2019 Aug 16;10:1046. doi: 10.3389/fphys.2019.01046 (PMC6707384; doi:10.3389/fphys.2019.01046)
Supplement: Supplementary file 1 [file Table_1.docx]

Supplementary data tables (to be made available online only and not included in the published paper)

1. Average effect sizes over time for muscle volumes
2. Average effect sizes over time for muscle cross sectional areas
3. Average effect sizes over time for torques and strength
4. Average effect sizes over time for contractile work capacity
5. Average effect sizes over time for muscle thickness
6. Average effect sizes over time for peak power
7. Average effect sizes over time for muscle activity
8. Average effect sizes over time for MVC during one rep max
9. Average effect sizes over time for power
10. Average effect sizes over time for performance based

Table 1 Average effect sizes over time for muscle volumes

|  | **Days** | | | | | | | | | | | |  |
| --- | --- | --- | --- | --- | --- | --- | --- | --- | --- | --- | --- | --- | --- |
|  | **7** | **14** | **20** | **27** | **28** | **29** | **35** | **42** | **55** | **56** | **57** | **89** | **90** |
| Plantar  Fexor Muscles | -0.3 1.1^32^ | **-1.0** 1.0^11,12^ | **-0.4** 1.3^4,56^ | **-0.9** 1.0^58,59^ | **-1.8** 1.1^11,12^ | **-1.2** 1.0^5,75^ | -1.7 1.1^53^ | **-2.2** 1.2^11,12^ | **-1.3** 1.0^58,59^ | **-3.2** 1.4^11,12^ | -2.6 1.3^75^ | -1.6 1.1^5^ |  |
| Dorsi Flexor  Muscles |  | **-0.4** 0.9^11, 12^ | **-0.1** 1.2^1,4,52^ | **-0.4** 1.0^58,59^ | **-0.5**  0.9^11, 12^ |  | -**0.5**  0.9^53^ | **-0.8**  1.0^11, 12^ | **-0.7**  1.0^58,59^ | **-2.8**  1.3^11, 12^ |  |  |  |
| Quadriceps  Muscles |  | **-1.3**  1.12^11,12^ | **-0.5**  1.3^1,3,4^ | -**0.7**  1^58,59^ | -**1.7**  1.2^11,12^ | **-0.8**  1^5,75^ | **-0.6**  0.9^53^ | **-2.2**  1.32^11,12^ | **-1.1**  1.1^7,58,59^ | **-2.8**  1.4^11,12^ | -1.3  1.1^75^ | **-1.4**  1^5^ |  |
| Hamstring  Muscles |  | **-1.1**  1.1^11,12^ | **-0.5**  1.3^1,3,4^ | **-0.5**  1^58,59,57^ | **-1**  1.1^11,12^ |  |  | **-1.8**  1.2^11,12^ | **-1.1**  1^58,59,57^ | **-2.2**  1.3^11,12^ |  |  |  |
| Hip Adductor Muscles |  | **-0.6**  1.1^11,12^ | **1.2**  1.5^3,4^ | **-0.2**  1^58,59^ | **-0.7**  1.1^11,12^ |  |  | **-1.1**  1.1^11,12^ | **-0.4**  1^58,59^ | **-1.1**  1.1^11,12^ |  |  |  |
| Gluteal Muscles |  |  |  | **-0.6**  1^57^ |  |  |  |  | **-0.7**  1^57^ |  |  |  |  |
| Hip Flexor Muscles |  |  |  |  |  |  |  |  |  |  |  |  | **-0.3**  0.9^15^ |
| OtherLower Limb Muscles | -0.2  1.2^32^ | **-0.7**  1^12^ | -0.5  1.3^1,3,4^ | **-0.8**  1.1^58,59,57^ | -0.3  1^12^ |  |  | **-0.6**  1^12^ | **-0.3**  0.9^58,59,57^ | **-0.9**  1.1^12^ |  |  |  |
| Erector Spinae Muscles |  |  |  | **0.3**  0.9^17^ |  |  |  |  | **0.3**  0.9^17^ |  |  |  | **-0.5**  0.9^15^ |
| Multifidus Muscle |  |  |  |  |  |  |  |  |  |  |  |  | **-0.7**  1^15^ |
| Other Trunk Muscles |  |  |  | **0.4**  0.9^17^ |  |  |  |  | **0.4**  0.9^17^ |  |  |  | **-0.2**  0.9^15,41^ |

Bold is effect size, underneath is 95% confidence interval with superscript cross reference to study ID in characteristics of analysed studies table

Table 2 Average effect sizes over time for muscle cross sectional areas

|  | **Days** | | | | | | | | | | | | | | | | | | | |
| --- | --- | --- | --- | --- | --- | --- | --- | --- | --- | --- | --- | --- | --- | --- | --- | --- | --- | --- | --- | --- |
|  | **2** | **7** | **9** | **10** | **14** | **16** | **20** | **21** | **27** | **28** | **29** | **30** | **35** | **37** | **42** | **47** | **55** | **56** | **60** | **89** |
| Plantar Fexor Muscles | -0.1 1.2^29^ |  | -0.1 1.2^29^ |  |  | -0.1 1.2^29^ | -0.3 1.3^1,2^ | -0.8 1.1^23^ |  | **-2.2** 1.2^70,71^ | **-0.2** 1.2^29^ | **-0.9** 1.0^21,25^ | **-0.6** 1.1^20,54^ |  |  |  |  |  | **-1.8** 1.2^64^ | **-4.5** 1.8^70,71^ |
| Dorsi Flexor Muscles |  |  |  |  |  |  | -**0.2**  1.4^1^ |  |  |  |  | **-1.3** 0.9^21^ | **-0.3** 0.9^54^ |  |  |  |  |  |  |  |
| Quadriceps Muscles | **-0.1** 1.2^29^ |  | **-0.1** 1.2^29^ |  | -**0.7**  1.0^61^ | **-0.1**  1.2^29^ | **-0.4** 1.4^1^ | **-0.5**  1.1^23^ |  | **-1.3**  1.0^61^ | **-0.1** 1.2^29^ |  | -**0.7**  1.3^20^ | **-1.1**  1.1^19^ | **-1.5**  1.1^61^ |  | **-1.5**  1.0^64^ | -1.9  1.1^61,64^ |  |  |
| Hamstring Muscles |  |  |  |  |  |  | **-0.4**  1.4^1^ |  |  |  |  |  |  |  |  |  |  |  |  |  |
| Hip Adductor Muscles |  |  |  | **-0.1**  0.9^43^ |  |  | **-0.2**  0.9^43^ |  |  |  |  |  |  |  |  |  |  |  |  |  |
| Gluteal Muscles |  |  |  |  |  |  |  |  |  |  |  |  | **-0.2**  1.2^20^ |  |  |  |  |  |  |  |
| Hip Flexor Muscles |  |  |  |  |  |  |  | **0.2** 1.1^16^ | **0.0**  0.9^13,14^ |  |  |  |  |  |  |  | **0.0**  0.9^13,14^ |  | **-0.2**  1.0^42^ |  |
| Other Lower Limb Muscles |  |  |  |  |  |  | **-0.6**  1.4^1^ |  |  |  |  | **-1.2**  1.2^25,21^ |  |  |  | **-1.0**  1.1^33^ |  |  |  |  |
| Erector Spinae Muscles |  |  |  |  |  |  |  | **0.6**  1.1^16^ | **0.8**  1.0^13,14^ |  |  |  |  |  |  |  | **-1.0**  1.0^13,14^ | **-1.1**  0.9^10^ | **-1.1**  1.0^42^ |  |
| Multifidus Muscle |  | **-0.4**  1.0^18^ |  |  | **-0.4**  1.0^18^ |  |  | **-0.5**  1.1^16^ | **-0.5** 0.9^13,14^ | **-0.6** 1.0^18^ |  |  |  |  | **-0.3**  1.0^18^ |  | **-0.7**  1.0^13,14^ | **-0.7**  1.4^18,10^ | **-1.1**  1.1^42^ |  |
| Other Trunk Muscle |  |  |  |  |  |  |  | **-0.1**  1.0^16^ | **-0.3**  0.9^13,14^ |  |  |  |  |  |  |  | **-0.4** 0.9^13,14^ |  | **-1.0** 1.1^42^ |  |
| Upper Limb Muscles |  |  |  |  |  |  |  |  |  | **-0.9** 1.0^70,71^ |  |  |  |  |  |  |  |  |  | **-1.4** 1.1^70,71^ |

Bold is effect size, underneath is 95% confidence interval with superscript cross reference to study ID in characteristics of analysed studies table

Table 3 Average effect sizes over time for torques and strength

|  | **Days** | | | | | | | | | | | | | | | | |
| --- | --- | --- | --- | --- | --- | --- | --- | --- | --- | --- | --- | --- | --- | --- | --- | --- | --- |
|  | **4** | **7** | **10** | **14** | **17** | **20** | **21** | **24** | **26** | **28** | **30** | **38** | **42** | **55** | **56** | **60** | **90** |
| Plantar Fexor Muscles |  |  |  | **-0.3** 1.0^31,8^ | **0.1** 1.0^74^ | **-0.4** 1.1^2^ | **-0.2** 1.1^23^ |  |  | **-0.4** 1.0^72^ | **-0.7** 0.8^68^ |  |  | **-1.7** 1.1^64^ |  | **-1.7** 0.7^55^ | **-2.0** 1.1^5,60^ |
| Dorsi Flexor Muscles |  |  |  |  |  |  |  |  |  | **-0.3** 1.0^72^ | **-0.6** 0.8^68^ |  |  |  |  | **-0.9** 0.6^55^ | **-0.3** 0.8^60^ |
| Quadriceps Muscles | **-0.2** 0.9^65^ | **-0.3** 0.9^65^ | **-0.3** 0.9^65^ | **-0.8** 1.0^31^ | **-0.4** 0.9^65^ | **-0.7** 1.4^1^ | **-0.3** 0.7^23^ | **-0.6** 0.9^65^ |  | **-0.5** 1.1^37,72^ | **-0.6** 1.1^27^ | **-0.9** 0.9^65^ | **-2.2** 1.3^19^ | **-2.0** 1.1^64^ | **-1.0** 0.9^62,65^ | **-1.7** 1.12^55,31^ | **-3.2** 1.4^5,60,6^ |
| Hamstring Muscles | **0.0** 0.9^61^ | **-0.1** 0.9^61^ | **0.0** 0.9^61^ |  | **-0.1** 0.9^61^ |  |  | **-0.4** 0.9^61^ |  | **-0.4** 1.0^72^ | **-0.2** 1.1^27^ | **-0.7** 1.0^61^ |  |  | **-0.8** 1.0^61^ | **-1.7** 0.7^55^ | **-0.5** 0.8^60^ |
| Hip Extensor Muscles |  |  |  |  |  |  |  |  |  |  |  | **-0.1** 0.9^53^ |  |  |  |  |  |
| Hip Flexor Muscles |  |  |  |  |  |  |  |  |  |  |  | **-0.1** 0.9^53^ |  |  |  |  |  |
| Other Trunk |  |  |  |  |  |  |  |  |  |  |  |  |  |  |  | **-2.2** 1.5^42,30^ | **-0.5** 0.9^15,60^ |
| Upper Limb Muscles |  |  | **-0.1** 1.2^39^ |  |  |  |  |  | **0.1** 1.2^39^ |  | **-0.4** 0.7^56^ | **-0.1** 0.9^53^ |  |  |  |  |  |

Bold is effect size, underneath is 95% confidence interval with superscript cross reference to study ID in characteristics of analysed studies table

Table 4 Average effect sizes over time for contractile work capacity

|  | **Days** |
| --- | --- |
|  | **14** |
| Plantar Flexors | -0.6 1.0^8^ |
| Quadricpes | **-0.6** 0.9^31^ |

Bold is effect size, underneath is 95% confidence interval with superscript cross reference to study ID in characteristics of analysed studies table

Table 5 Average effect sizes over time for muscle thickness

|  | **Days** | | | | | |
| --- | --- | --- | --- | --- | --- | --- |
|  | **7** | **14** | **26** | **35** | **42** | **56** |
| Plantar Fexor Muscles |  |  |  | **-1.4** 1.0^26^ |  |  |
| Dorsi Flexor Muscles |  |  |  | **-0.9** 0.9^26^ |  |  |
| Quadriceps Muscles |  |  |  | **-1.8** 1.0^26^ |  |  |
| Erector Spinae Muscles | **-0.5** 1.0^18^ | **-0.5** 1.0^18^ | **-0.5** 1.0^18^ |  | **-0.5** 1.0^18^ | **-0.5** 1.0^18^ |
| Internal Oblique Muscle |  | **-0.9** 1.0^18^ | **-0.9** 1.0^18^ |  | **-0.8** 1.0^18^ | **-0.8** 1.0^18^ |
| Transversus Abdominis Muscle |  | **-1.6** 1.1^18^ | **-1.4** 1.1^18^ |  | **-1.4** 1.1^18^ | **-1.4** 1.1^18^ |
| Upper Limb  Muscles |  |  |  | **0.9** 0.9^26^ |  |  |

Bold is effect size, underneath is 95% confidence interval with superscript cross reference to study ID in characteristics of analysed studies table

Table 6 Average effect sizes over time for peak power

|  | **Days** | | | |
| --- | --- | --- | --- | --- |
|  | **56** | **60** | **62** | **90** |
| Plantar Fexor Muscles |  |  | **-1.6** 1.1^75^ | -2.1 1.2^5^ |
| Quadriceps  Muscles |  |  | **-1.4** 1.1^75^ | -1.9 1.1^5^ |
| Jump | **-1.6** 1.0^22^ | **-2.3** 1.2^6^ |  |  |

Bold is effect size, underneath is 95% confidence interval with superscript cross reference to study ID in characteristics of analysed studies table

Table 7 Average effect sizes over time for EMG muscle activity

|  | Days | | | | | | | | |
| --- | --- | --- | --- | --- | --- | --- | --- | --- | --- |
|  | 14 | 15 | 20 | 21 | 30 | 45 | 55 | 56 | 90 |
| Plantar  Fexor  Muscles | **0.0** 1.0^8^ |  | **0.4** 1.2^52,73^ | **-0.2** 1.0^64^ |  |  | **-0.2** 1.0^64^ | **0.2** 0.9^22^ | -0.9 1.0^5^ |
| Dorsi Flexor  Muscles | **-0.5** 1.2^8,24^ |  | **0.5** 1.1^52^ |  |  |  |  |  |  |
| Quadriceps  Muscles | **-0.5** 1.4^24^ |  | **0.3** 1.2^3,73^ | **-0.1** 0.9^64^ |  |  | **-0.1** 0.9^64^ |  | **-0.9** 1.0^5^ |
| Erector Spinae Muscles |  |  |  | **-0.3** 1.1^16^ |  |  |  |  |  |
| Mutifidus  Muscle |  |  |  | **-0.2** 1.1^16^ |  |  |  |  |  |
| Hip Flexor  Muscles |  |  |  | **-0.2** 1.1^16^ |  |  |  |  |  |
| Shoulder  Muscles |  | **-0.6** 1.0^34^ |  |  | **-0.5** 1.0^34^ | **-0.3** 1.0^34^ |  |  |  |
| Forearm  Muscles |  | **-0.7** 1.0^34^ |  |  | **-0.8** 1.0^34^ | **-0.6** 1.0^34^ |  |  |  |

Bold is effect size, underneath is 95% confidence interval with superscript cross reference to study ID in characteristics of analysed studies table

Table 8 Average effect sizes over time for MVC during one rep max

|  |  | Days | | | | | | | | | | | | | |  |  |  |
| --- | --- | --- | --- | --- | --- | --- | --- | --- | --- | --- | --- | --- | --- | --- | --- | --- | --- | --- |
|  | 6 | 7 | 10 | 13 | 14 | 15 | 17 | 20 | 26 | 28 | 30 | 35 | 45 | 56 | 60 | 62 | 90 | 120 |
| Plantar Fexor Muscles |  | **-0.7** 1.2^45,49^ |  |  | -1.1 1.1^8^ |  | **-0.3** 1.0^66^ | **-0.4** 1.1^73^ |  |  |  |  |  | **-0.8** 0.9^22^ | **-1.4** 1.3^50^ | **-2.1** 1.3^75^ | **-1.7** 1.1^69,75^ | **-2.0** 1.5^44,46,47,48,51^ |
| Dorsi Flexor Muscles |  |  |  |  | -0.1 1.0^8^ |  |  |  |  |  |  |  |  |  | **-0.5** 0.6^55^ |  |  |  |
| Quadriceps Muscles |  | **-0.5** 1.3^35^ |  |  | **-0.7** 1.3^35^ |  |  | **-0.8** 1.3^3,35,73^ |  | **-0.3** 1.0^72^ |  | **-1.4** 1.4^20^ |  |  | **-1.1** 0.6^55^ | **-1.5** 1.1^75,36^ | -2.4 1.2^5^ |  |
| Hamstring Muscles |  | **-0.4** 1.3^35^ |  |  | **-0.8** 1.3^35^ |  |  | **-1.1** 1.3^35^ |  | **-0.6** 1.0^72^ |  |  |  |  |  |  |  |  |
| Hip Flexor Muscle |  | **-0.9** 1.3^35^ |  |  | **-1.0** 1.3^35^ |  |  | **-1.0** 1.3^35^ |  |  |  |  |  |  |  |  |  |  |
| Other lower limb | **-0.3** 1.2^39^ |  |  | **-0.6** 1.3^39^ |  |  |  | **-0.6** 1.3^39^ |  | **-0.5** 1.3^39^ |  | **-0.9** 1.3^20^ |  |  |  |  | **-1.5** 1.0^6^ |  |
| Other upper limb |  | **0.0** 1.2^35^ | **0.2** 1.2^39^ |  | **-0.1** 1.2^35^ | **-0.2** 1.0^34^ |  | **-0.5** 1.3^35^ | **-0.1** 1.2^39^ |  | **-0.0** 1.0^34^ | **-0.0** 1.2^20^ | **-0.0** 1.0^34^ |  |  |  |  |  |

Bold is effect size, underneath is 95% confidence interval with superscript cross reference to study ID in characteristics of analysed studies table

Table 9 Average effect sizes over time for power

|  | Days | | |
| --- | --- | --- | --- |
|  | 7 | 14 | 20 |
| Plantar Fexor Muscles |  | -0.5 1.0^8^ |  |
| Quadricep Muscles | **-0.6** 1.3^35^ | **-0.9** 1.3^35^ | **-1.7** 1.5^35^ |
| Hamstring Muscles | **-0.1** 1.2^35^ | **-0.4** 1.2^35^ | **-0.6** 1.3^35^ |
| Hip Flexor Muscles | **-0.3** 1.2^35^ | **-0.5** 1.3^35^ | **-0.6** 1.3^35^ |
| Other upper limb | **-0.0** 1.2^35^ | **-0.2** 1.2^35^ | **-0.6** 1.3^35^ |

Bold is effect size, underneath is 95% confidence interval with superscript cross reference to study ID in characteristics of analysed studies table

Table 10 Average effect sizes over time for performance based

|  | Days | | | | | | | |
| --- | --- | --- | --- | --- | --- | --- | --- | --- |
|  | 14 | 30 | 42 | 44 | 56 | 60 | 62 | 90 |
| Endurance | **-1.1** 1.1^38^ | **-1.2** 0.9^68^ |  |  |  |  |  |  |
| Jumping |  |  | **-0.6** 1.1^19^ | **-2.0** 1.3^33^ | **-1.2** 1.0^22^ | **-2.3** 1.3^36^ |  |  |
| Sit to stand |  |  |  |  |  | **-1.1** 1.0^36^ |  |  |
| Balance |  |  |  |  |  | **1.2** 0.8^60^ |  | **1.1** 0.8^60^ |
| Sprint time |  |  |  |  |  |  | **2.1** 1.1^36^ |  |

Bold is effect size, underneath is 95% confidence interval with superscript cross reference to study ID in characteristics of analysed studies table
